# Diagnostic performance of HScore for macrophage activation syndrome complicating Still’s disease in adults: a multicentre case–control study

**DOI:** 10.1093/rheumatology/keag473

**Published:** 2026-08-31

**Authors:** Toshihiko Shiga, Yuji Nozaki, Saki Tsuchimoto, Hirotaka Yamazawa, Chisato Ashida, Daisuke Tomita, Tetsu Itami, Shoichi Hino, Masafumi Sugiyama, Yasuyoshi Morita, Koji Kinoshita, Shinya Rai

**Affiliations:** Department of Haematology and Rheumatology, Kindai University School of Medicine, Osaka, Japan; Department of Haematology and Rheumatology, Kindai University School of Medicine, Osaka, Japan; Department of Rheumatology, Kindai University Nara Hospital, Nara, Japan; Department of Haematology and Rheumatology, Kindai University School of Medicine, Osaka, Japan; Department of Haematology and Rheumatology, Kindai University School of Medicine, Osaka, Japan; Department of Haematology and Rheumatology, Kindai University School of Medicine, Osaka, Japan; Department of Haematology and Rheumatology, Kindai University School of Medicine, Osaka, Japan; Department of Rheumatology, Izumi City General Hospital, Osaka, Japan; Department of Rheumatology, Kindai University Nara Hospital, Nara, Japan; Department of Haematology and Rheumatology, Kindai University School of Medicine, Osaka, Japan; Department of Haematology and Rheumatology, Kindai University School of Medicine, Osaka, Japan; Department of Haematology and Rheumatology, Kindai University School of Medicine, Osaka, Japan

**Keywords:** HScore, macrophage activation syndrome, haemophagocytic lymphohistiocytosis, Still’s disease, adult-onset Still’s disease, HLH-2004 diagnostic criteria, 2016 EULAR/ACR/PRINTO classification criteria, diagnostic performance

## Abstract

**Objectives:**

To validate the diagnostic performance of the HScore for Still’s disease (SD)-associated macrophage activation syndrome (MAS; SD-MAS) in adults compared with those of the HLH-2004 diagnostic criteria used with modifications (HLH-04) and the 2016 EULAR/ACR/PRINTO classification criteria for systemic juvenile idiopathic arthritis-associated MAS (MAS-2016).

**Methods:**

This multicentre case–control study, conducted at three medical centres in Japan between 2004 and 2023, enrolled patients ≥16 years with active SD and allocated them into SD without MAS and SD-MAS groups by an expert panel. For each patient, we calculated the HScore, HLH-04, MAS-2016 and modified HScore, which excluded haemophagocytosis on bone marrow aspirate. Receiver operating characteristic curve analysis was used to assess the discriminative ability of each criterion. Logistic regression analysis was performed separately for ‘model-1’ (HScore and HLH-04) and ‘model-2’ (MAS-2016 and modified HScore).

**Results:**

This study included 86 patients with SD, 25 of whom had SD-MAS. The HScore showed the best area under the curve at 0.991 (95% CI 0.977–1.000), with a cut-off value of 192 (sensitivity, 96.0%; specificity, 96.7%). The optimal cut-off for the modified HScore was 171 (sensitivity, 92.0%; specificity, 83.6%). Multivariate analysis identified only the HScore in model-1 [odds ratio (OR) 1.271; 95% CI 1.006–1.606], whereas both MAS-2016 (OR 4.249; 95% CI 1.636–11.041) and modified HScore (OR 1.066; 95% CI 1.017–1.118) were identified in model-2.

**Conclusions:**

The HScore and modified HScore could be useful tools for diagnosing SD-MAS in adults with and without bone marrow aspiration, respectively.

## Graphical Abstract

**Graphical Abstract:**
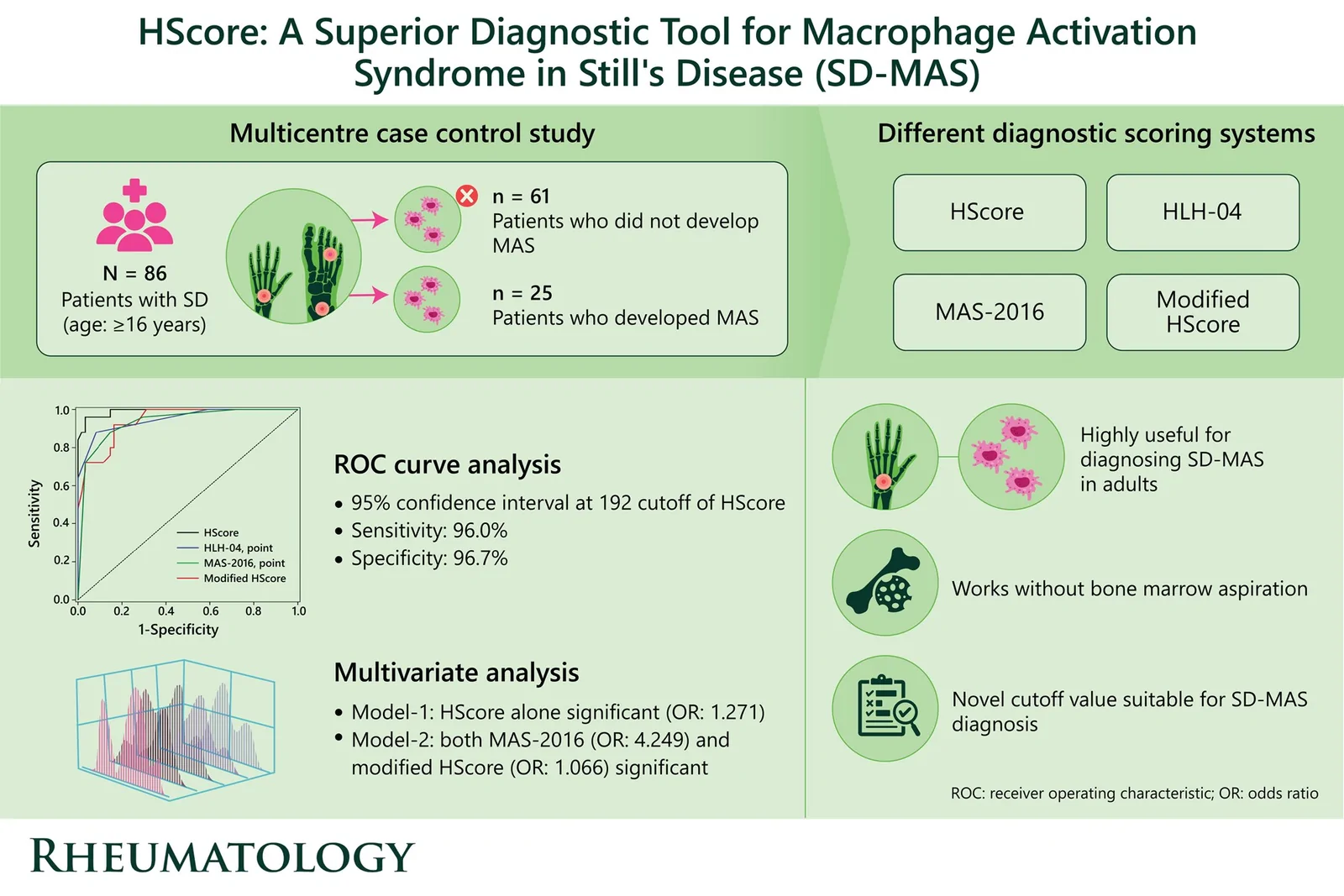

Rheumatology key messages

- The HScore demonstrated excellent diagnostic performance for Still’s disease–associated macrophage activation syndrome (SD-MAS) in adults.
- The modified HScore retained good diagnostic performance without bone marrow aspiration.
- A higher HScore cut-off value than the original 169 may better identify adult SD-MAS.

## Introduction

Adult-onset Still’s disease (AOSD) and systemic JIA (sJIA) have recently been recognized as the same disease on the basis of several lines of evidence [1, 2]. Moreover, it has been proposed that the name of the diseases be unified into ‘Still’s disease (SD)’ [1, 2]. SD is a rare multisystemic autoinflammatory disease of uncertain aetiology, characterized by manifestation of clinical symptoms such as high spiking fever, evanescent rash and arthritis. It can sometimes cause macrophage activation syndrome (MAS), a subtype of haemophagocytic lymphohistiocytosis (HLH) secondary to rheumatic diseases [3, 4]. MAS was first reported in the 1980s in cases of severe systemic sJIA (i.e. SD in children) [5–7]; however, it is an equally serious life-threatening complication in patients with AOSD (i.e. SD in adults) [8]. Diagnosing SD-associated MAS (SD-MAS) as quickly and accurately as possible is important, because MAS represents the major refractory and life-threatening complication of SD [1, 9–11]. However, no globally agreed upon criteria exist for diagnosis and classification of SD-MAS in all age groups at the present time [12, 13].

The original HLH-2004 diagnostic criteria (HLH-04), proposed by Henter *et al.* [14], are among the criteria used for diagnosing SD-MAS. However, these criteria have several limitations. The HLH-04 has been developed specifically in paediatric populations to diagnose familial or primary HLH and has never been prospectively validated for reactive or secondary HLH in adults [15]. Moreover, some criteria (e.g. molecular diagnosis or NK cell activity) cannot be measured in routine practice. These limitations have made directly applying the HLH-04 to diagnose adult HLH and MAS difficult, including in patients with SD.

The MAS-2016 criteria (MAS-2016) were developed to classify sJIA-associated MAS (sJIA-MAS; i.e. SD-MAS in children) by the EULAR/ACR/PRINTO collaborative initiative group [16, 17]. Several studies have tested whether the MAS-2016 can also be used to identify AOSD-associated MAS (AOSD-MAS; i.e. SD-MAS in adults) [18–20]. However, the MAS-2016 is still not widely used in SD practice across all age groups, as concerns about the differences between adult and paediatric diseases have not been fully addressed.

The HScore, published by Fardet *et al.* is a scoring system that assesses nine parameters derived from clinical and laboratory findings in a stratified manner and was developed primarily for the diagnosis of reactive or secondary HLH in adults [21]. However, the majority of the underlying conditions for HLH in this study were haematological malignancies or infections; SD-MAS in adults was present in only 1.3% of cases. To date, very few studies have evaluated the diagnostic utility of the HScore, focusing only on SD-MAS in adults [22, 23]. Therefore, it is unclear whether the HScore could be used for diagnosing SD-MAS in adults.

Thus, we conducted the present study to compare the diagnostic performance of the HScore with those of the HLH-04 used with modifications or MAS-2016 for SD-MAS in adults.

## Methods

### Study design and patients

This multicentre, observational, case–control study retrospectively analysed the medical records of adults with Still’s disease from three different medical centres between April 2004 and March 2023: Kindai University School of Medicine and Izumi City General Hospital in Osaka, and Kindai University Nara Hospital in Nara, all in Japan. We searched for data of all patients admitted to each centre during the time period defined above, with International Classification of Diseases 10 codes for ‘Adult-onset Still’s disease (M061)’, ‘Adult Still’s disease (M0610)’ or ‘Still’s disease (M0820)’. We included patients aged ≥16 years at the time of SD diagnosis and excluded those with a previous diagnosis of JIA. We defined conventional AOSD as ‘SD in adults’ in this study. All patients had a fever of ≥38°C for at least 1 week at the clinically active stage of disease with SD-associated symptoms. We assessed the disease activity in patients with SD by a modified Pouchot score and defined cases of scores of >4 as those of clinically active disease [24]. The patients fulfilled Yamaguchi’s diagnostic criteria after appropriate exclusion of febrile diseases, such as infections, malignancies, and other rheumatic diseases [25]. This study complied with the standards of the Declaration of Helsinki and the Strengthening the Reporting of Observational Studies in Epidemiology (STROBE) guidelines [26], and was approved by the Ethics Committees of Kindai University School of Medicine and Kindai University Nara Hospital (approval no. R05-027) and Izumi City General Hospital (approval no. 23-J06). Opt-out informed consent was obtained, as this was a non-invasive, non-interventional, retrospective study using only existing information from medical records without collecting new samples, in accordance with the Ethical Guidelines for Medical and Health Research Involving Human Subjects in Japan.

### Data collection

**Figure 1:**
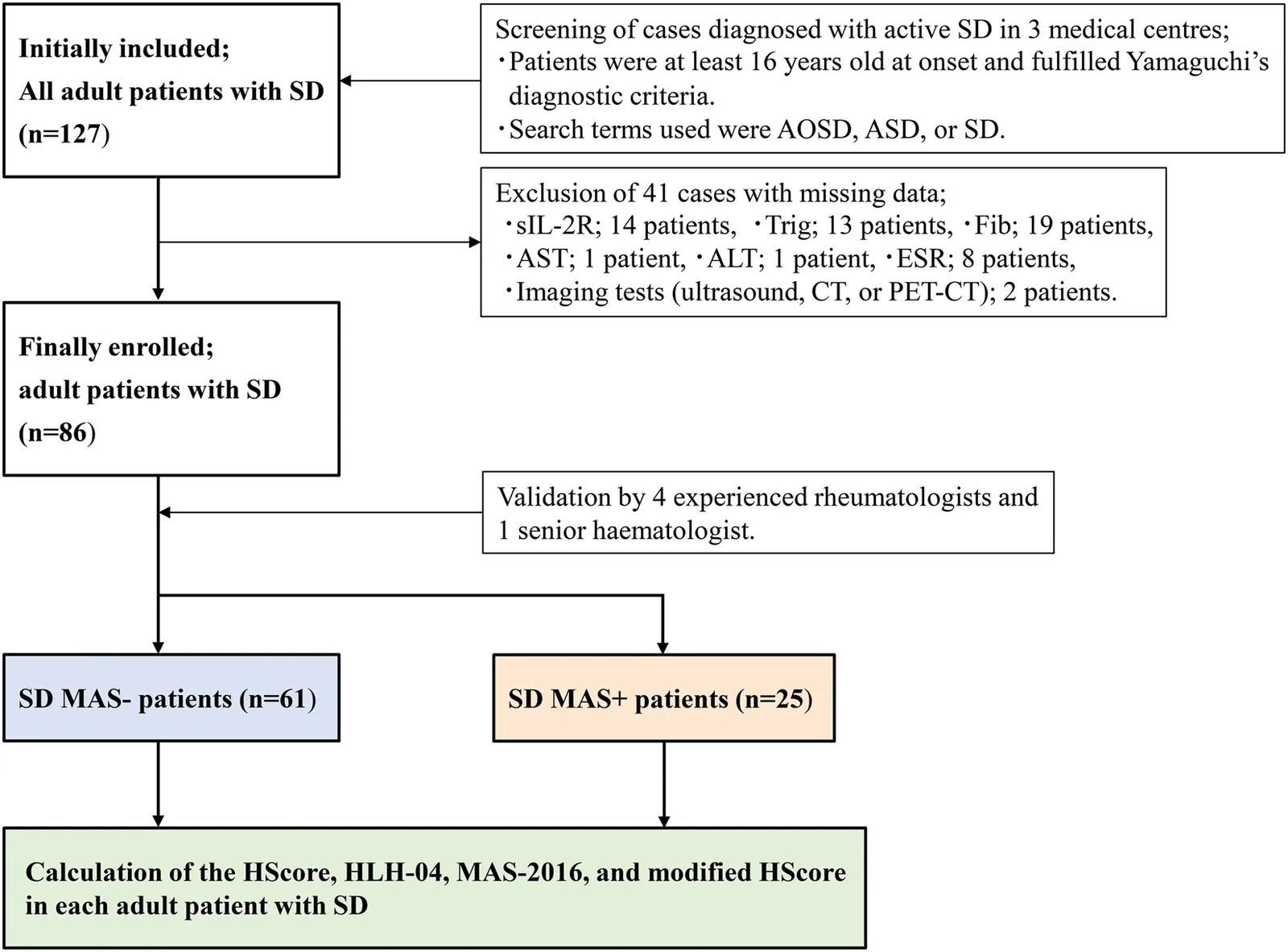
Flowchart of patient registration. Patients were identified at three medical centres: Kindai University School of Medicine, Kindai University Nara Hospital and Izumi City General Hospital from April 2004 to March 2023. AOSD: adult-onset Still’s disease; ASD: adult Still’s disease; SD: Still’s disease; ALT: alanine transaminase; AST: aspartate transaminase; Fib: fibrinogen; sIL-2R: soluble IL-2 receptor; Trig: triglyceride

We analysed demographic data, such as age and sex; clinical features, such as fever, maximum body temperature, skin rash, arthralgia or arthritis, myalgia, sore throat and abdominal pain; and laboratory findings, including ferritin levels, soluble IL-2 receptor (sIL-2R) levels, blood cell counts, triglyceride (Trig) levels, fibrinogen (Fib) levels, and aspartate aminotransferase (AST) levels, as summarized in Tables 1 and 2. In addition, alanine aminotransferase (ALT), CRP, ESR, and lactate dehydrogenase were also collected as supplementary laboratory variables to comprehensively characterize the clinical and laboratory features of patients with SD and SD-MAS but were not used in the calculation of the HScore, HLH-04 or MAS-2016 criteria. Ultrasonography, CT and positron emission CT were performed to assess the presence of: organomegaly, such as lymphadenopathy and hepatosplenomegaly; serositis, such as pericarditis and pleurisy; and parenchymal lung lesions, such as those of interstitial pneumonia. From the medical records, we checked whether all patients had undergone bone marrow aspiration and, if they had, whether haemophagocytosis was present. A history of immunosuppressive therapy, including glucocorticoids, immunosuppressive drugs, and biologic DMARDs, before diagnosis was also identified. All data were evaluated at the time of SD diagnosis or SD-MAS in adults. If no assessment was recorded on that day, the acceptable time range for each parameter was extended according to the protocol (Table 1). As illustrated in Fig. 1, patients with even one missing data point (regarding clinical characteristics, laboratory findings or imaging tests) were excluded from this study.

**Table 1:** Data collection of parameters for the HScore, HLH-04 and MAS-2016.

Parameters	The acceptable time range for each parameter if not assessed at the date of diagnosis of SD in adults or SD-MAS
Fever	Highest body temperature that could be confirmed from the onset of SD in adults or SD-MAS until diagnosis
Non-fever clinical symptoms^a^	Symptoms that could be confirmed from the onset of SD in adults or SD-MAS until diagnosis
Ferritin, ng/ml	Max within ±3 days
Blood cell counts^b^	Min within ±3 days
AST, IU/l	Max within ±3 days
Trig, mg/dl	Max within ±5 days
Fib, mg/dl	Min within ±5 days
sIL-2R, U/ml	Max within ±10 days
Other blood laboratory data^c^	Max within ±3 days
Organomegaly^d^	±30 days
Bone marrow aspirate	±30 days
Pre-existing immunosuppression	Immunosuppressive therapies used for at least 30 days before diagnosis of SD in adults or SD-MAS

a Skin rash, arthralgia/arthritis, myalgia, sore throat.

b White blood cell count, haemoglobin, platelet count.

c Alanine transaminase; CRP; ESR; l-lactate dehydrogenase.

d Lymphadenopathy, splenomegaly, hepatomegaly. AST, aspartate transaminase; Fib, fibrinogen; Max, maximum value; Min, minimum value; sIL-2R, soluble IL-2 receptor; Trig, triglyceride; SD-MAS: Still’s disease–associated macrophage activation syndrome.

**Table 2:** Demographic, clinical and laboratory characteristics of the SD-MAS − and MAS + groups.

Parameters	SD-MAS− (*n* = 61)	SD-MAS+ (*n* = 25)	*P*-value
Age, years (IQR)	56 (35.5–66)	58 (43.5–70)	0.34
Sex: female, *n* (%)	47 (77.0)	21 (84.0)	0.57
Clinical features			
Fever, *n* (%)			
≥38.0°C	61 (100)	25 (100)	
<38.4°C	6 (9.8)	1 (4.0)	0.67
38.4–39.4°C	27 (44.3)	8 (32.0)	0.34
>39.4°C	28 (45.9)	16 (64.0)	0.15
Skin rash, *n* (%)	53 (86.9)	23 (92.0)	0.72
Arthralgia/arthritis, *n* (%)	53 (86.9)	17 (68.0)	0.065
Myalgia, *n* (%)	16 (26.2)	8 (32.0)	0.60
Sore throat, *n* (%)	39 (63.9)	17 (68.0)	0.80
Lymphadenopathy, *n* (%)	37 (60.7)	21 (84.0)	0.044
Splenomegaly, *n* (%)	36 (59.0)	20 (80.0)	0.083
Hepatomegaly, *n* (%)	13 (21.3)	11 (44.0)	0.062
Laboratory data, median (IQR)			
Ferritin, ng/ml	5354 (2102–13 500)	49 470 (25 702–73 104)	<0.0001
sIL-2R, U/ml	1524 (1028–2092)	3978 (2383–5550)	<0.0001
ESR, mm/h	73 (56–86)	48 (23–66)	0.0002
CRP, mg/dl	8.3 (4.7–15.6)	13.5 (4.8–20.2)	0.28
LDH, IU/l	470 (380–659)	1109 (891–1432)	<0.0001
AST, IU/l	54 (35–82)	117 (79–226)	<0.0001
ALT, IU/l	37 (21–60)	50 (28–123)	0.14
WBC, counts/µl	11 380 (8560–14 130)	9400 (6230–15 460)	0.23
Neutrophils, counts/µl	9505 (6262–12 949)	6934 (4982–13 918)	0.23
Lymphocytes, counts/µl	963 (702–1286)	947 (393–1597)	0.66
Hb, g/dl, median (IQR)	11.2 (9.5–12.8)	9.7 (9.1–12.7)	0.28
PLT × 10^4^, count/µl, median (IQR)	29.8 (23.1–36.6)	11.3 (7.8–15.0)	<0.0001
Trig, mg/dl, median (IQR)	138 (99–165)	185 (162–256)	<0.0001
Fib, mg/dl, median (IQR)	471 (367–587)	219 (177–279)	<0.0001
Bone marrow aspirate, *n* (%)	6 (9.8)	22 (88.0)	<0.0001
Haemophagocytosis in bone marrow, *n* (%)	0/6 0	20/22 (90.9)	<0.0001
Immunosuppressive therapies before diagnosis, *n* (%)	13 (21.3)	7 (28.0)	0.58
HScore, score, median (IQR)	131 (102–168)	234 (211–259)	<0.0001
modified HScore, score, median (IQR)	131 (102–168)	200 (183–227)	<0.0001
HLH-04; measured score, median (IQR)	3 (2–3)	5 (4–6)	<0.0001
HLH-04; criteria (5/7 or more) fulfilled, *n* (%)	0 (0)	16 (64.0)	<0.0001
MAS-2016: measured score, median (IQR)	2 (1–3)	5 (4–5)	<0.0001
MAS-2016: criteria fulfilled, *n* (%)	18 (29.5)	24 (96.0)	<0.0001

Data are numbers (with percentages) or medians [interquartile ranges (IQR)]. ALT: alanine transaminase; AST: aspartate transaminase; Fib: fibrinogen; Hb: haemoglobin; LDH: l-lactate dehydrogenase; PLT: platelet count; SD-MAS: Still’s disease–associated macrophage activation syndrome; sIL-2R: soluble Il-2 receptor; Trig: triglyceride; WBC: white blood cell; HLH-04: modified HLH-2004 diagnostic criteria (≥5/7); MAS-2016: 2016 EULAR/ACR/PRINTO classification criteria for systemic juvenile idiopathic arthritis-associated macrophage activation syndrome.

### Calculation of the HScore, HLH-04 and MAS-2016

Using the data obtained, we determined the HScore, HLH-04 and MAS-2016 in each patient (Supplementary Table S1). The HScore was calculated as the total score for the nine variables as specified in the original article [21]. In addition, we defined ‘modified HScore’ as the HScore excluding ‘haemophagocytosis features on bone marrow aspirate’. In this study, we used the HLH-04 criteria with modifications, defined as fulfilment of five or more of seven non-molecular/non-genotypic criteria after excluding ‘low or absent NK-cell activity’, as recently proposed by Henter *et al.* [27]. For consistency, this modified version is referred to as ‘HLH-04’ throughout the manuscript. The MAS-2016 requires item 1 and at least two items among 2–5: (item 1) ferritin level >684 ng/ml, (item 2) platelet count ≤181 × 10^9^/l, (item 3) AST level >48 U/l, (item 4) Trig level >156 mg/dl and (item 5) Fib level ≤360 mg/dl) [16, 17] and we summed the measured scores as for the HLH-04. However, the total score was set to 0 points for patients who did not meet the mandatory requirements of item 1.

### Definition of SD-MAS in adults

Each of the three authors (Y.N., M.S. and S.H.), all experienced rheumatologists, reviewed the medical records of all enrolled patients with SD and extracted those of patients with SD-MAS. They were blinded to each other when classifying the SD-MAS in adults; the diagnosis was confirmed before calculating the HScore, HLH-04 and MAS-2016 scores. During the SD-MAS assessment, they were not provided with details of the components of each criterion shown in Supplementary Table S1. If the attending physicians in charge on admission suspected complications of MAS in patients with SD and performed bone marrow aspirations, which revealed haemophagocytosis features, the cases were preferentially classified as SD-MAS in adults. We excluded cases of MAS due to other causes, such as infections, malignancy, other inflammatory or autoimmune disorders, and drug-induced cytopaenia. In particular, cases of MAS occurring after the administration of tocilizumab, the first biologic DMARD approved in Japan for the treatment of AOSD, were excluded among those of SD-MAS in adults [28–30]. Once all patients had been assessed by the three authors, the principal investigator (T.S.) and an experienced haematologist (Y.M.) joined the consultation to compare and validate their results. In cases where a bone marrow aspiration had not been performed, or had shown no evidence of haemophagocytosis, the five experts discussed whether the diagnosis of SD-MAS could be made based on a comprehensive assessment of the patient’s clinical course, such as a tendency for blood cell counts to decrease rapidly over a short period. Finally, cases wherein all five investigators agreed upon the diagnosis of SD-MAS in adults were classified into the SD-MAS + group and all other patients were assigned to the SD-MAS − group.

### Statistical analysis

Continuous variables are expressed as medians (interquartile ranges), while categorical variables are expressed as numbers (*n*) and percentages (%). The Mann–Whitney *U* test or Fisher’s exact test was used to compare demographic characteristics, clinical features and laboratory findings between the SD MAS − and MAS + groups. We performed receiver operating characteristic (ROC) curve analysis to determine the best prediction accuracy of all classification criteria for differential diagnosis between the SD-MAS − and SD-MAS + groups with reference to the HScore, HLH-04, MAS-2016 and modified HScore. In addition, we calculated the area under the curve (AUC), cut-off value, sensitivity and specificity. The Youden Index was used to calculate each cut-off value. A logistic regression analysis was performed to identify more useful diagnostic criteria that could be used to differentiate the SD-MAS − group from the SD-MAS + group. Using multivariate analysis, we analysed ‘model-1’ separately for the criteria including bone marrow aspiration (the HScore and HLH-04) and ‘model-2’ for the criteria not including it (MAS-2016 and modified HScore). We calculated the concordance index (c-index) to assess which of the nine parameters comprising the HScore contributed to its utility in the diagnosis of SD-MAS. Statistical significance was set at *P* < 0.05. Statistical analyses were performed using the SPSS Statistics ver. 22.0 (IBM Japan, Ltd, Tokyo, Japan) and GraphPad Prism 9 (GraphPad Software, San Diego, CA, USA).

## Results

### Study population and characteristics of the SD-MAS − and SD-MAS + groups

A total of 127 adults with SD were included in this study. However, after excluding patients with missing data, 86 patients were finally enrolled in this study, with 61 patients with SD without MAS (SD-MAS − group) and 25 with SD-MAS (SD-MAS + group), as detailed in the flowchart shown in Fig. 1. Table 2 summarizes the demographic characteristics, clinical features and laboratory data of the SD-MAS − and SD-MAS + groups. There were no statistically significant differences between the two groups in age, sex or most clinical characteristics. Fever was assessed according to the HScore definition, stratified into three categories (<38.4°C, 38.4–39.4°C and >39.4°C) without significant differences in any of the categories. However, there was significantly more lymphadenopathy in the SD-MAS + group (*P* = 0.044) and a trend toward increased splenomegaly (*P* = 0.083) and hepatomegaly (*P* = 0.062). Although there was no statistically significant difference in the occurrence of arthralgia/arthritis between the two groups, an upward trend was evident in the SD-MAS − group (*P* = 0.065). The platelet count was the only blood cell count that differed significantly between the two groups (*P* < 0.0001). The SD-MAS + group had significantly higher ferritin, sIL-2R, AST and Trig levels and lower Fib levels than the SD-MAS − group (*P* < 0.0001). Bone marrow aspiration was performed in 22 of the 25 patients in the SD-MAS + group, and haemophagocytosis was confirmed in 20 patients. Three patients did not undergo bone marrow aspiration because one refused the test and the other two had problems with the hospital laboratory system such as the closure due to extended holidays. In contrast, only six patients in the SD-MAS − group underwent the procedure, none of whom had haemophagocytosis.

Before the diagnosis, 12 (19.8%) patients in the SD-MAS − group and 7 (28.0%) in the SD-MAS + group received glucocorticoids. Other immunosuppressants (ciclosporin: *n* = 1, MTX: *n* = 1) were administered only to the SD-MAS − group. None of the patients in this study tested positive for the HIV.

The SD-MAS + group achieved significantly higher scores for all criteria: HScore, HLH-04, MAS-2016 and modified HScore (*P* < 0.0001). Among the patients in the SD-MAS + group, 36.0% and 4.0% did not meet the HLH-04 (≥5/7) and MAS-2016 criteria, respectively.

### Identification of more useful diagnostic criteria that differentiate between the SD-MAS − and SD-MAS + groups

**Figure 2:**
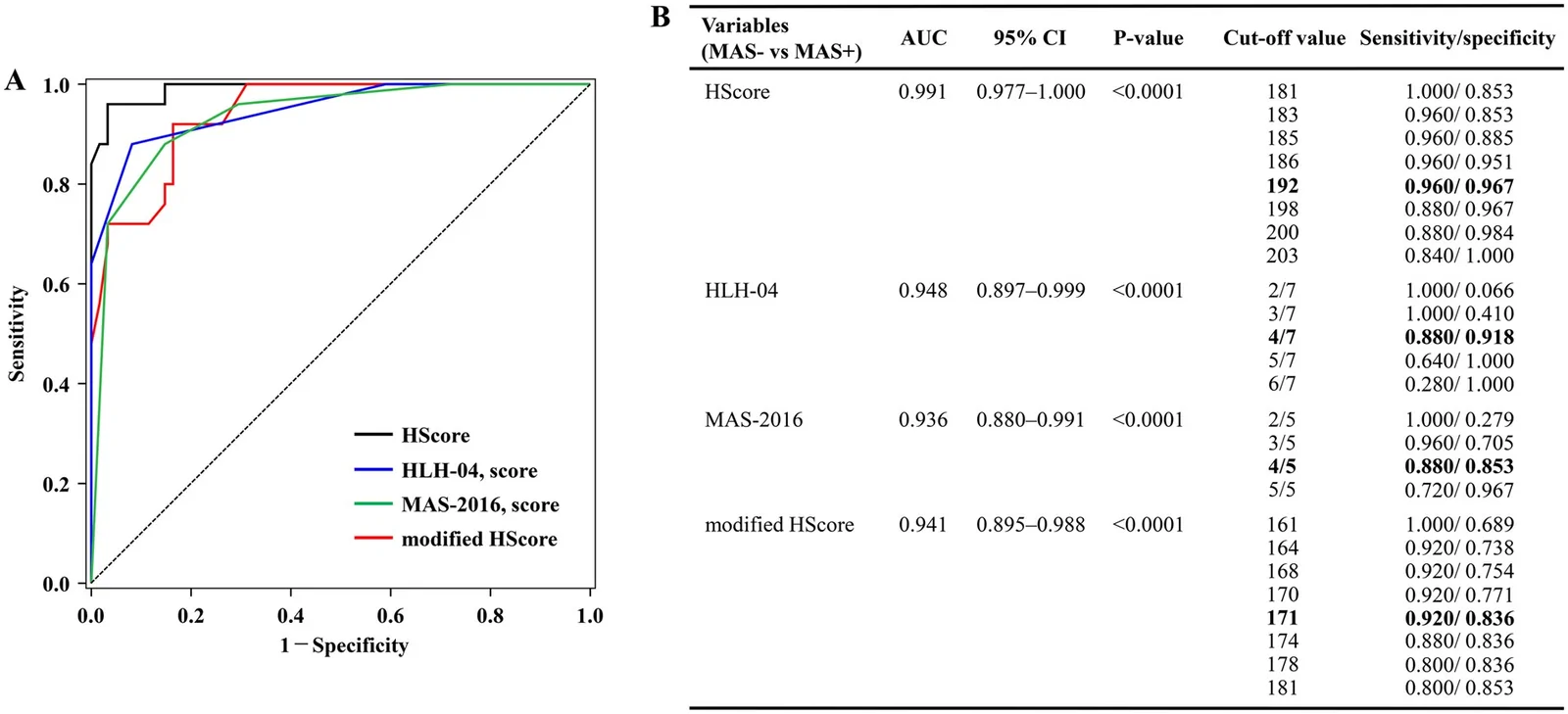
ROC curve analysis for differential diagnosis between the SD-MAS − and SD-MAS + groups based on the HScore, HLH-04, MAS-2016 and modified HScore. (A) The predictive performance of each diagnostic criterion of the HScore, HLH-04, MAS-2016 and modified HScore was validated using the ROC curve analysis. (B) The ability of differential diagnosis was indicated by the AUC and 95% CI. Cut-off values for the HLH-04 and MAS-2016 are shown as the number of fulfilled criteria/items over the total number of criteria/items. AUC: area under the curve; MAS: macrophage activation syndrome; HLH-04: modified HLH-2004 diagnostic criteria (5/7); MAS-2016: 2016 EULAR/ACR/PRINTO classification criteria for systemic juvenile idiopathic arthritis-associated macrophage activation syndrome; ROC: receiver operating characteristic; SD-MAS: Still’s disease–associated macrophage activation syndrome

We performed ROC curve analyses to differentiate between the SD-MAS − and SD-MAS + groups with reference to four diagnostic criteria: HScore, HLH-04, MAS-2016 and modified HScore (Fig. 2A and 2B). The results showed that among these criteria, the HScore had the best AUC of 0.991 (95% CI 0.977–1.000, *P* < 0.0001), and the AUC of the modified HScore was 0.941 (95% CI 0.895–0.988, *P* < 0.0001), which was slightly better than that for the MAS-2016 (0.936). The best predictive accuracy for the discrimination of MAS was achieved with a cut-off value of 192 for the HScore, 4/7 for the HLH-04 and 171 for the modified HScore, with details of the sensitivity and specificity of the criterion shown in Fig. 2B. In addition, the MAS-2016 had better sensitivity at the 3/5 cut-off value (sensitivity, 96.0% and specificity, 70.5%) and superior specificity at the 4/5 cut-off value (sensitivity, 88.0% and specificity, 85.3%). We then performed logistic regression analysis to identify more useful diagnostic criteria for differentiating SD-MAS (Table 3). Using multivariate analysis, only the HScore was extracted in model-1 for criteria that included bone marrow aspirate [odds ratio (OR) 1.271, 95% CI 1.006–1.606, *P* = 0.044], whereas both the MAS-2016 and modified HScore were identified in model-2 for criteria that did not include bone marrow aspirate (MAS-2016: OR 4.249, 95% CI 1.636–11.041, *P* = 0.003; modified HScore: OR 1.066, 95% CI 1.017–1.118, *P* = 0.008).

**Table 3:** Univariable and multivariable analyses to identify the useful criteria to differentiate patients with SD-MAS.

Variables	Univariable analysis		Multivariable analysis			
			Model-1		Model-2	
	OR (95% CI)	*P*-value	OR (95% CI)	*P*-value	OR (95% CI)	*P*-value
Age, years	1.015 (0.986–1.044)	0.320				
Sex; female	1.564 (0.460–5.321)	0.474				
HScore, score (per 1)	1.202 (1.058–1.366)	0.005	1.271 (1.006–1.606)	0.044		
HLH-04, score (per 1)	19.486 (5.029–75.501)	<0.0001				
MAS-2016, score (per 1)	6.093 (2.923–12.703)	<0.0001			4.249 (1.636–11.041)	0.003
modified HScore, score (per 1)	1.085 (1.041–1.131)	0.0001			1.066 (1.017–1.118)	0.008

Age and sex were adjusted in the multivariable analyses. Model-1 was analysed for the criteria including bone marrow aspiration (HScore and HLH-04) and model-2 for the criteria not including it (MAS-2016 and modified HScore). SD-MAS: Still’s disease–associated macrophage activation syndrome; HLH-04: modified HLH-2004 diagnostic criteria (5/7); MAS-2016: 2016 EULAR/ACR/PRINTO classification criteria for systemic juvenile idiopathic arthritis-associated macrophage activation syndrome; OR: odds ratio.

### Factors that contributed most to the differentiation of SD-MAS in adults among the nine parameters included in the HScore

Based on the results of the ROC curve and multivariate analyses, we calculated the c-index to assess which of the nine parameters included in the HScore contributed the most to the differentiation of SD-MAS (Table 4). The results showed that the highest contribution was made by ‘haemophagocytosis features on bone marrow aspirate’ (c-index: 0.900, 95% CI 0.805–0.995), followed by ‘Fib’ (c-index: 0.824, 95% CI 0.708–0.939) and then ‘Ferritin’ (c-index: 0.763, 95% CI 0.665–0.861).

**Table 4:** C-index to determine the HScore components contributing to the differentiation of patients with SD-MAS.

**Parameters**	C-index	95% CI
Known underlying immunosuppression^a^: no (0)/yes (18)	0.544	0.405–0.682
Temperature (°C): <38.4 (0)/38.4–39.4 (33)/>39.4 (49)	0.600	0.472–0.729
Organomegaly: no (0)/splenomegaly or hepatomegaly (23)/splenomegaly and hepatomegaly (38)	0.653	0.523–0.784
Number of cytopaenias^b^: 1 lineage or less (0)/2 lineages (24)/3 lineages (34)	0.612	0.471–0.753
Ferritin (ng/ml): <2000 0/2000–6000 (35)/>6000 (50)	0.763	0.665–0.861
Trig (mg/dl): <133 0/133–354 (44)/>354 (64)	0.724	0.615–0.832
Fib (mg/dl): ≤250 (30)/>250 (0)	0.824	0.708–0.939
AST (IU/l): <30 (0)/≥30 (19)	0.582	0.457–0.707
Haemophagocytosis features on bone marrow aspirate: no (0)/yes (35)	0.900	0.805–0.995

a HIV positive or receiving long-term immunosuppressive therapy.

b Defined as a leucocyte count of ≤5000/µl and/or a haemoglobin level of ≤9.2 g/dl and/or a platelet count of ≤110 000/µl. Numbers in brackets () indicate points for the HScore criteria scoring. SD-MAS: Still’s disease–associated macrophage activation syndrome; C-index: concordance index; AST: aspartate transaminase; Fib: fibrinogen; Trig: triglyceride.

## Discussion

This was a multicentre case–control study in Japan investigating the diagnostic performance of the HScore compared with those of the HLH-04 or MAS-2016 to differentiate MAS complicating SD in adult patients. To the best of our knowledge, few similar studies have been conducted where the target disease is limited to SD in adults [22, 23]. The results of our analyses demonstrated that the HScore showed high diagnostic accuracy for differentiating SD-MAS in adults, and that the modified HScore retained diagnostic utility when bone marrow aspiration was not performed. With the changing definitions of SD, there is still no gold standard applicable to all ages for classifying and diagnosing MAS, a serious complication of SD [31, 32]. Therefore, we suggest that re-evaluating whether the existing diagnostic and classification criteria for HLH or MAS could be used for SD-MAS in adults is crucial.

Previously, Batu *et al.* reported the assessment of the HScore for reactive haemophagocytic syndrome in 94 patients with rheumatic diseases, including 69 (73.4%) patients with SD: AOSD, 46 (48.9%) and sJIA, 23 (24.5%). The authors found that when applying the HScore to patients with reactive haemophagocytic syndrome, the best cut-off value was 190.5 with a sensitivity of 96.7% and a specificity of 98.4% [22]. This cut-off was very close to the optimal cut-off identified in the present study (192), which yielded a sensitivity of 96.0% and a specificity of 96.7%. Importantly, the similarity between these cut-off values, despite differences in the study populations, supports the potential reproducibility of a higher HScore cut-off for diagnosing SD-MAS in adults.

These HScore cut-offs (192 in the present study and 190.5 in the study by Batu *et al.* [22]) were higher than the original cut-off value of 169 reported by Fardet *et al.* [21] (sensitivity, 93%; specificity, 86%). This might be due to differences in the pathogenesis of the underlying diseases causing HLH or MAS. A comparative study of SD-MAS and other secondary HLH/MAS [lymphoma-associated hemophagocytic syndrome (LAHS) or non-LAHS] in adults showed that the level of serum ferritin, a parameter of the HScore, is significantly higher in patients with SD-MAS [33]. In addition, a multicentre cohort study by Bilston *et al.* validated the HScore and HLH-04 for the diagnosis of HLH with different underlying causes, including inflammatory disorders (20.2%). In this study, the HScore showed better sensitivity than did the HLH-04 for diagnosing inflammatory HLH (i.e. MAS), as opposed to HLH due to other causes, and further improved its diagnostic accuracy when its cut-off value was set above 204 [34]. These previous findings and the results of the present study suggest that the cut-off value of the HScore for the diagnosis of SD-MAS in adults should be modified from that of the original.

A disadvantage of both the HScore and HLH-04 is that they include bone marrow aspiration according to their criteria. For various reasons such as patient refusal, bone marrow aspiration is not always possible in routine clinical practice. In addition, haemophagocytosis is not always evident in the bone marrow aspirate of patients with SD-MAS, particularly in the early stages of the disease [35, 36]. Conversely, the MAS-2016 is widely used for classifying sJIA-MAS and does not require bone marrow aspiration. Although several studies have reported the usefulness of this classification for diagnosing SD-MAS in adults [18–20], its specificity may be reduced in adult populations because the mandatory ferritin threshold (>684 ng/ml), originally established in sJIA-MAS, may be insufficiently specific for adult SD, in which marked hyperferritinemia is frequently observed even in patients without MAS [37]. Whether an adult-specific ferritin threshold could further improve the diagnostic performance of the MAS-2016 criteria in adults remains to be determined in independent validation cohorts. Nevertheless, in the present study, the MAS-2016 score showed clinically useful overall discriminative performance for identifying SD-MAS in adults, with high sensitivity at the 3/5 cut-off and improved specificity at higher cut-off values. We created a modified HScore by omitting bone marrow aspiration from the original version and verified its diagnostic accuracy. This demonstrated that the diagnostic performance of the modified HScore was comparable to that of the MAS-2016.

In this study, the c-index was calculated to determine which of the nine parameters in the HScore contributed most to the differentiation of SD-MAS. The presence of phagocytosis in bone marrow aspiration is undoubtedly important for the HScore (c-index: 0.900; 95% CI 0.805–0.995), although the Fib level was identified as the next most important parameter (c-index: 0.824; 95% CI 0.708–0.939). Activated macrophages secrete ferritin and plasminogen activator factors. This leads to increased plasma concentration and excessive fibrinolysis [38–40]. However, SD-MAS might be characterized by a higher frequency of Fib levels of <250 mg/dl than HLH/MAS due to other factors. Regular measurement of Fib and ferritin levels during the daily management of SD may help in the early diagnosis of MAS.

Our study has some limitations. First, this was a retrospective study involving a small number of patients with rare diseases and the data were collected by reviewing hospital medical records. Consequently, some form of information bias might have existed. For instance, the ‘body temperature’ or ‘fever’ criterion in each classification was based on the highest recorded value in the medical records. However, these values did not always accurately reflect the maximum body temperature during the clinical course. In addition, this study defined SD-MAS after consulting multiple specialists. However, this definition does not completely rule out the subjectivity among individual evaluators, which might have introduced a selection bias. Furthermore, as the expert panel diagnosis of SD-MAS in this study includes clinical information that overlaps with the components of the HScore, a certain degree of incorporation bias is unavoidable. Nevertheless, in the absence of a gold standard diagnostic criterion for SD-MAS, this is considered a reasonably acceptable alternative method for including target patients in a study. Finally, when scoring the MAS-2016, we assigned a score of zero to cases that did not meet the mandatory criterion of ‘ferritin >684 ng/ml’. Although this might have influenced the statistical evaluation, only one case fell into this category in practice.

## Conclusions

The HScore demonstrated excellent diagnostic performance for SD-MAS in adults when bone marrow aspirate findings were available. The modified HScore also retained good diagnostic performance without bone marrow aspiration. A higher HScore cut-off may be appropriate for SD-MAS in adults; however, external validation in larger prospective cohorts is required before clinical implementation and to determine whether these findings apply to paediatric SD-MAS.

## Supplementary Material

keag473_Supplementary_Data

## Data Availability

The underlying data for this article will be shared upon reasonable request from the corresponding author.
