# Supplementary material for: Diagnostic performance of HScore for macrophage activation syndrome complicating Still’s disease in adults: a multicentre case–control study
Source: Rheumatology (Oxford). 2026 Aug 31;65(9):keag473. doi: 10.1093/rheumatology/keag473 (PMC13570798; doi:10.1093/rheumatology/keag473)
Supplement: Supplementary file 1 [file keag473_supplementary_data.docx]

| Supplemental Table S1. Comparison of the diagnostic criteria evaluated in the present study | | | | | | | | |  |  |  |  |  |  |  |
| --- | --- | --- | --- | --- | --- | --- | --- | --- | --- | --- | --- | --- | --- | --- | --- |
| **Criteria** |  |  | **HScore** |  |  |  |  | **HLH-04** |  |  |  | **MAS-2016** |  |  |  |
|  |  |  |  |  |  |  |  |  |  |  |  |  |  |  |  |
| Clinical features | |  |  |  |  |  |  |  |  |  |  |  |  |  |  |
| Fever |  |  | <38.4℃ (0), 38.4–39.4℃ (33), >39.4℃ (49) | | | |  | ≥38.5℃ (Yes/No) | |  |  | Yes/No |  |  |  |
|  |  |  |  | | | |  |  | |  |  |  |  |  |  |
| Organomegaly | |  | Splenomegaly or Hepatomegaly (23) | | | |  | Splenomegaly (Yes/No) | | |  | - |  |  |  |
|  |  |  | Splenomegaly and Hepatomegaly (38) | | | |  |  |  |  |  |  |  |  |  |
|  |  |  | Neither (0) |  |  |  |  |  |  |  |  |  |  |  |  |
| Laboratory data | |  |  |  |  |  |  |  |  |  |  |  |  |  |  |
| Cytopaenia |  |  | Number of cytopenias; | | |  |  | At least 2 cell lineages (Yes/No) | | |  | Platelets ≤181,000/µL (Yes/No) | | |  |
|  |  |  | 1 lineage or less (0)/2 lineages (24)/3 lineages (34) | | | | | Neutrophils <1,000/µL,  Haemoglobin <9.0g/dL, | | | |  |  |  |  |
|  |  |  | Leukocytes ≤5,000/µL,  Haemoglobin ≤9.2g/dL, | | | |  | Platelets <100,000/µL. | |  |  |  |  |  |  |
|  |  |  | Platelets ≤110,000/µL | |  |  |  |  |  |  |  |  |  |  |  |
| Ferritin, ng/mL | |  | <2,000 (0), 2,000–6,000 (35), >6,000 (50) | | | |  | ≥500 (Yes/No) | |  |  | >684 (Yes/No) | |  |  |
| Triglyceride, mg/dL | |  | <133 (0), 133–354 (44), >354 (64) | | |  |  | Triglycerides ≥265 or Fibrinogen ≤150 | | | | >156 (Yes/No) | |  |  |
| Fibrinogen, mg/dL | |  | ≤250 (30), >250 (0) | |  |  |  | (Yes/No) | |  |  | ≤360 (Yes/No) | |  |  |
| Aspartate transaminase, IU/L | | | <30 (0), ≥30 (19) | |  |  |  | - |  |  |  | >48 (Yes/No) | |  |  |
| Soluble IL-2Ra (CD25), U/mL | | | - |  |  |  |  | ≥2,400 (Yes/No) | |  |  | - |  |  |  |
| Haemophagocytosis | |  | No (0), Yes (35) | |  |  |  | Yes/No |  |  |  | - |  |  |  |
| Low/ absent NK-cell activity | | | - |  |  |  |  | Excluded |  |  |  | - |  |  |  |
| Known immunosuppression | | | No (0), Yes (18) | |  |  |  | - |  |  |  | - |  |  |  |
| Fulfilment of criteria | |  | Calculate the total score for the nine parameters; | | | | | Five or more of seven criteria | | | | Febrile patients with known or suspected sJIA, | | | |
|  |  |  | Scores of >169 are 93% sensitive and 86% specific | | | | |  | | | | Ferritin >684 ng/mL and two or more | | |  |
|  |  |  | for adult HLH/MAS in the original study [21] | | | |  |  |  |  |  | additional items | |  |  |
| The point system of the HScore; see http://saintantoine.aphp.fr/score/. | | | | | |  |  |  |  |  |  |  |  |  |  |
| HLH: haemophagocytic lymphohistiocytosis, MAS: macrophage activation syndrome. sJIA: systemic juvenile idiopathic arthritis. HLH-04: modified HLH-2004 diagnostic criteria (five of seven criteria after excluding the low or absent NK-cell activity criterion), | | | | | | | | | | | | | | |  |
| MAS-2016: 2016 EULAR/ACR/PRINTO classification criteria for sJIA-MAS | | | | | | |  |  |  |  |  |  |  |  |  |
